# Correlation analysis of the risk of ischemic stroke with related risk factors in a health examination population

**DOI:** 10.12669/pjms.40.11.9563

**Published:** 2024-12

**Authors:** Xue Bai, Hui Wang, Jiangzhe Li, Jinjin Xu, Pan Cai

**Affiliations:** 1 Xue Bai Department of Physical Examination, Baoding No.1 Central Hospital, Baoding 071000, Hebei, China; 2 Hui Wang Department of Urological, Baoding No.1 Central Hospital, Baoding 071000, Hebei, China; 3 Jiangzhe Li Western Medicine Pharmacy Static Dispensing Center, Baoding No.1 Central Hospital, Baoding 071000, Hebei, China; 4 Jinjin Xu Department of Geriatric, Baoding No.1 Central Hospital, Baoding 071000, Hebei, China; 5 Pan Cai Department of Rheumatology and Immunology, Baoding No.1 Central Hospital, Baoding 071000, Hebei, China

**Keywords:** Health Examination, Stroke, High-Risk Population, Risk Factor Analysis

## Abstract

**Objective:**

To explore the correlation between the risk of ischemic stroke and related risk factors in a health examination population.

**Methods:**

This was a retrospective study. A total of 300 subjects undergoing health examination in the physical examination center of Baoding NO.1 Central Hospital were selected from January 2023 to December 2023, and divided into the normal group (Group-N) and the risk group according to the criteria of cerebral hemodynamic integral value(CVHI). The risk group was further subdivided into three groups, including the high-risk group(Group-H), the medium-risk population(Group-M), and the low-risk group(Group-L), with 75 subjects in each group. The general data, including BMI, smoking, and the incidence of hypertension, diabetes, and hyperlipidemia, were analyzed and compared between the two groups.

**Results:**

Multivariable logistic regression analysis was conducted using stroke risk factors as the dependent variables after assignment, and it was found that BMI, smoking, hypertension, diabetes, hyperlipidemia, and hyperuricemia were independent risk factors for ischemic stroke (P<0.05); the incidence of obesity and overweight, smoking, hypertension, hyperlipidemia, and hyperuricemia were significantly increased in Group-H compared with those in groups M, L, and N, with statistically significant differences(P<0.05). The chi square test showed statistically significant differences in the stroke risk factors among different age groups (P<0.05).

**Conclusion:**

The incidence of ischemic stroke risk factors varies among different age groups in the health examination population, and is related to poor lifestyle and underlying diseases.

## INTRODUCTION

Stroke is a group of diseases that occur when brain tissue is damaged due to sudden rupture of cerebral blood vessels in the brain or cerebral ischemia caused by vascular occlusion.^1^ There are two types of strokes, namely ischemic stroke and hemorrhagic stroke. Stroke is characterized by high morbidity, high disability, high mortality and high recurrence rates, and listed as the top three diseases with the highest mortality along with coronary heart disease and malignant tumors. Ischemic stroke, the most common type of this disease, accounts for more than 80% of all stroke cases^2^, with extremely high mortality and disability rates.

Studies have found that the incidence of ischemic stroke has been increasing in recent years, making it a public health problem worldwide that threatens human health and life.^3^ About 30% of patients with stroke die, and 70% of survivors live with disabilities such as hemiplegia and aphasia.^4^ Stroke seriously endangers the lives, health, and quality of life(QoL) of the general population, bringing a heavy burden to patients, their families, and society, and has become a major public health issue affecting human health. The etiology and pathogenesis of stroke have not been not fully, scientifically and convincingly explained to date. There are no effective and satisfactory treatment options for existing stroke, and it is even difficult to influence the natural history and outcomes of this disease.^5^ Stroke is a disease caused by various factors, including living style and environment. The main risk factors include sex, age, family history, obesity, smoking, alcohol drinking and physical inactivity, and sex, age and family history among others are non-modifiable factors.^6^

As a disease commonly encountered in neurosurgery, stroke is globalized and has become a public health problem worldwide that threatens the health of middle-aged and elderly people due to its high morbidity, disability and mortality. With the development of society and economy, the acceleration of the pace of life, and the increased pressure at work in young people, the incidence of this disease in young population is increasing, which is of serious concern for clinicians.^7^ Investigation on risk factors is the basic and primary task in disease prevention, and established risk factors are helpful to guide the implementation of disease prevention measures. Current studies have found that the combined effect of multiple factors is a risk factor for stroke, and active prevention and intervention of stroke significantly reduce the risk of this disease.The present study was designed to explore the risk factors that affect the development of stroke in healthy people by analyzing the clinical data in individuals undergoing health examination to provide a theoretical basis for the prevention and control of stroke.

## METHODS

This was a retrospective study. Three hundred subjects undergoing health examination in the physical examination center of Baoding No.1 Central Hospital were selected from January 2023 to December 2023. They were divided into the normal group (Group-N) and the risk group according to the criteria of cerebral hemodynamic integral value (CVHI). The risk group was further subdivided into three groups, including the high-risk group (Group-H), the medium-risk population (Group-M), and the low-risk group (Group-L), with 75 subjects in each group. Subject data included the medical data of veterans from Baoding No.1 Central Hospital were selected information and management system, collected their various information of all subjects.

### Ethical Approval:

The study was approved by the Institutional Ethics Committee of Baoding No.1 Central Hospital (No.:2023103; date: October 23, 2023), and written informed consent was obtained from all participants. The general demographic data of this population are shown in Table-I.

**Table-I:** Comparison of general data between the normal group and the risk group (*χ̅*±*S*).

Items	The normal group	The risk group	t/χ^2^	P
n	75	225		
Age(years)	63.27±11.32	64.05±10.42	0.39	0.70
Sex (male, %)	48(64%)	130(58%)	0.76	0.38
BMI (kg/m^2^) *	21.84±1.08	28.35±3.66	13.21	0.00
Hypertension (n, %) *	12(16%)	60(26%)	27.56	0.00
Smoking (n, %) *	15(20%)	112(50%)	19.78	0.00
Drinking (n, %) *	18(24%)	86(38%)	4.58	0.03
Hyperlipidemia (n, %) *	13(17%)	126(56%)	32.81	0.00
Diabetes (n, %) *	7(9%)	58(26%)	10.01	0.00
Hyperuricemia (n, %) *	11(15%)	65(29%)	5.72	0.02

* P<0.05.

### Diagnostic Criteria:

Evaluation criteria for CVHI^8^: A cerebrovascular function testing instrument was used to detect bilateral common carotid arteries by a specialist physician, and a total of 10 parameters in three domains, including cerebrovascular blood supply, elastic characteristics and microcirculation, were collected. These parameters included average blood flow (Qmean), maximum blood velocity (Vmax), minimum blood velocity (Vmin), average blood velocity (Vmean), peripheral resistance (Rv), characteristic impedance(Zev), pulse wave velocity (Wv), dynamic resistance (DR), critical pressure level (Cp), and the difference between diastolic and critical pressure (Dp). The CVHI scores were calculated by a built-in calculation and analysis system of the testing instrument. CVHI scores ranged from 0 to 100 points. Subjects with a CVHI score of >75 points were considered normal in cerebrovascular function; and those with a CVHI score ≤75 points were considered as risk with abnormal cerebrovascular function and included in the risk group that was risky in stroke. Subjects in the risk group were further subdivided into three groups, including the high-risk group, with CVHI <25, the medium-risk group, with 25≤CVHI<50, and the low-risk group, with 50≤CVHI≤75.

Evaluation criteria for hypertension included systolic pressure ≥140 mmHg, and/or diastolic pressure ≥90 mmHg, and/or receiving medical treatment.^9^

Evaluation criteria for dyslipidemia were triglyceride (TG) ≥1.7 mmol/L, or total cholesterol (TC) ≥6.22 mmol /L, or low-density lipoprotein cholesterol (LDL-C) ≥4.14 mmol/L, or high-density lipoprotein cholesterol(HDL-C) <1.04 mmol/L or ≥1.55.^10^

Evaluation criteria for abnormal blood glucose included fasting blood glucose (GLU) ≥7.0 mmol/L, and abnormal blood glucose was considered even the GLU test was normal if the subjects had a history of diabetes and received non-drug or drug interventions.^11^

Evaluation criteria for hyperuricemia were uric acid (UA) >420 mmol/L (for males) and >360 mmol/L(for females), and hyperuricemia was determined even the serum UA test was normal if the subject had a history of hyperuricemia and received non-drug or drug interventions.^12^

Evaluation criteria for overweight and obesity: Body mass index (BMI) was used to classified subjects as normal, overweight and obesity^13^, i.e., normal: 18.5 kg/m^2^≤BMI<24 kg/m^2^; overweight: 24 kg/m^2^≤BMI<28 kg/m^2^; and obesity: BMI≥28 kg/m^2^.

### Study Procedure:

Medical records of the health examination population were collected, and the general clinical data, including sex, age, smoking, drinking, hypertension, diabetes, and hyperlipidemia, were analyzed and compared between the normal group and the risk group. The related risk factors for ischemic stroke in the health examination population were explored. Furthermore, the differences in sex, age, laboratory tests, and poor lifestyle were analyzed among groups with different risks of stroke to evaluate the correlation of different risk populations with the exposure levels of different stroke risk factors.

### Statistical Analysis:

All data were analyzed using SPSS 25.0 software. Measurement data were presented as (*χ̅*±*S*). Two independent sample t-test was used for comparisons between groups, and χ^2^ test was used for comparisons of rates. Logistic regression analysis was used for the correlation factors analysis, analysis of variance was used for comparisons of different risk factors, and t-test was used for pairwise comparisons. Differences with p<0.05 were considered statistically significant.

## RESULTS

Multivariable logistic regression analysis was conducted using stroke risk factors as the dependent variables after assignment, with α _enter_ =0.05 and α _output_ =0.10. It was found that BMI, smoking, hypertension, diabetes, hyperlipidemia, and hyperuricemia were independent risk factors for ischemic stroke (P<0.05) (Table-II).

**Table-II:** Logistic regression analysis of risk factors related to ischemic stroke in health examination population (*χ̅*±*S*) (n=300).

Factors	β	SE	Waldχ^2^	P	OR	95.0% CI
BMI*	3.235	0.430	2.538	0.001	3.218	3.170-3.316
Smoking*	3.537	0.544	3.120	0.001	3.076	2.837-3.308
Hypertension*	3.476	0.424	3.260	0.002	3.328	3.280-3.472
Diabetes*	3.206	0.417	4.053	0.001	4.129	3.830-4.347
Hyperlipidemia*	4.160	0.562	4.705	0.002	3.608	3.507-3.712
Hyperuricemia*	3.672	0.464	3.758	0.002	3.617	3.532-3.710
Age	2.847	2.363	1.453	0.361	1.826	1.713-2.038
Sex	3.460	2.504	2.108	0.264	2.413	2.270-2.662
Drinking	2.863	2.417	2.340	0.389	2.074	1.931-2.239

* P<0.05.

Detection of stroke risk factors in different populations suggested that the incidences of overweight or obesity, smoking, hypertension, hyperlipidemia, and hyperuricemia were significantly increased in Group-H compared with those in groups M, L, and N, with statistically significant differences (P<0.05) (Table-III).

**Table-III:** Detection of stroke risk factors in different groups (*χ̅*±*S*) (n=75).

Factors	Group-N	Group-L	Group-M	Group-H	χ^2^	P
Overweight or obesity*	6(8%)	13(17%)	22(29%)	34(45%)	9.74	0.00
Smoking*	15(20%)	26(35%)	37(49%)	49(65%)	10.32	0.00
Hypertension*	12(16%)	17(23%)	19(25%)	24(32%)	5.41	0.00
Diabetes	7(9%)	11(15%)	16(21%)	22(29%)	2.52	0.32
Hyperlipidemia*	13(17%)	24(32%)	32(43%)	70(93%)	13.07	0.00
Hyperuricemia*	11(15%)	15(20%)	23(31%)	27(36%)	4.76	0.01

* P<0.05.

Among the stroke risk factors in different age groups, a normal distribution was observed in the incidences of overweight, obesity, and hypertension, while the incidences of diabetes, hyperlipidemia, and hyperuricemia increased with age. The chi square test showed statistically significant differences in stroke risk factors among different age groups (P<0.05) (Table-IV).

**Table-IV:** Analysis of related stroke risk factors (*χ̅*±*S*).

Age	Overweight	Obesity	Hypertension	Diabetes	Hyperlipidemia	Hyperuricemia
≤40	6.32	3.68	1.06	0.33	2.32	1.54
40-49	13.47	10.82	3.21	1.27	5.63	12.07
50-69	38.52	10.76	15.37	11.83	7.68	23.62
≥70	30.13	5.37	10.37	8.98	7.24	20.39
χ^2^	9.84	5.73	14.58	12.35	6.08	13.06
P	0.00	0.00	0.00	0.00	0.00	0.00

*P<0.05.

## DISCUSSION

The development of ischemic stroke is influenced by the combined effect of various factors. The results of the present study showed that BMI, smoking, hypertension, diabetes, hyperlipidemia and hyperuricemia were independent risk factors for ischemic stroke. The adverse effect of poor lifestyle on health has been recognized by most people. According to Maida et al.,^14^ the incidence of obesity, smoking and excessive drinking is significantly higher in individuals at high risk of stroke than that in normal people, indicating that smoking and drinking are risk factors for ischemic stroke. Hypertension is an important risk factor for ischemic stroke. Studies have shown that the incidence of stroke is positively correlated with the incidence of hypertension, and the risk of stroke increases by two times for every increase of 10mmHg in diastolic pressure, and decreases by 35%-40% for every decrease of 5-7 mmHg in diastolic pressure.^15^ Studies have also found that the regional distribution of ischemic stroke cases is consistent with the incidence of hypertension, and the degree of blood pressure variation, disease duration, the type of hypertension directly affect the development of ischemic stroke.^16^

Effective stroke screening and prevention, and early detection and timely intervention of risk factors is an urgent and significant task to safeguard the health rights and interests of people. The stroke risk factors are very complex and can be divided into two categories, namely modifiable and non-modifiable risk factors.^17^ Modifiable risk factors include hypertension, diabetes, dyslipidemia, inappropriate diet and overnutrition, smoking, alcohol drinking, physical inactivity, obesity, and hyperuricemia. Mendelson et al.^18^ showed that effective primary, secondary and tertiary prevention measures targeting stroke risk factors can reduce the development of stroke, control the conditions of patients with existing stroke, and decrease the incidence, disability and mortality of stroke. Individuals at high risk of stroke are identified according to the criteria of stroke screening^19^, and strict health education and specific treatment targeting the modifiable risk factors are provided to prevent the development and recurrence of stroke. For individuals not at high risk of stroke, appropriate health instructions and risk factor intervention can prevent the development and progression of stroke risk factors and reduce the development of stroke. With the continuous improvement of living standards and cultural quality, people are paying more attention to the quality of life, and the concept has shifted to focus on disease prevention. Therefore, more individuals and organizations place high priority on health examination and believe it is an annual task that must be done.

The risk of diabetes, hyperlipidemia and heart disease is increasing with the change of lifestyle in recent years. Diabetes and hyperlipidemia are associated with altered blood patterns and increased blood viscosity, which increase heart burden and blood supply to distal tissues and organs, leading to ischemia and hypoxia of the affected tissues and organs and consequently the increased risk of ischemic stroke.^20^ At the same time, heart disease can lead to insufficient blood supply to the heart, which may reduce the effective circulating blood volume and induce ischemia and hypoxia in brain tissue, Finally the risk of ischemic stroke increases.^21^

It was also found in the present study that the incidences of obesity or overweight, smoking, hypertension, hyperlipidemia and hyperuricemia were significantly increased in the high-risk group compared with those in the medium- and low-risk groups and the normal group. This possible reasons behind this included that most of the individuals undergoing health examination in the present study lived in urban and rural areas, and did not have sufficient knowledge of poor lifestyles and health risk factors. Furthermore, the relative high labor intensity, lack of effective physical exercise, and inappropriate diet also resulted in overweight and dyslipidemia.^22^ In addition, studies have shown that abnormal conditions, such as diabetes, hyperlipidemia, and hyperuricemia, increase with age.

Therefore, different forms of health education should be provided in clinical practice to individuals undergoing health examination to promote the establishment of favorable lifestyles and correct understanding of the knowledge of stroke and its related risk factors and related prevention measures to consequently prevent the development and delay the progression of stroke.^23^ For health examination populations in different age groups, especially the elderly, personalized stroke management strategies should be developed according to the sex- and age-specific risk factors, and appropriate standardized management should be implemented to help reduce or delay the incidence of stroke and its complications.

### Limitations:

It includes a small sample size and limited outcome measures used. Future studies with larger sample sizes and more outcome and follow-up parameters will be conducted, and the outcome of high-risk individuals after drug intervention will be included to bring more benefits to patients.

## CONCLUSIONS

The incidence of stroke risk factors varies in different age groups. Active exploration of the risk factors affecting the development of ischemic stroke, implementation of stroke screening and prevention, early detection of high-risk individuals, close monitoring of the poor lifestyle and underlying diseases such as hypertension, diabetes and hyperlipidemia, and enforcement of early intervention can significantly reduce the morbidity and mortality of stroke in a high-risk population, which is of great significance for stroke prevention.

### Authors’ Contributions:

**XB** and **HW:** Carried out the studies, data collection, and drafted the manuscript.

**JL and JX:** Statistical analysis, participated in its design and critical review.

**PC:** Data collection and entering data in SPSS. Review.

All authors read, approved the final manuscript and are responsible and accountable for the accuracy or integrity of the work.
